# Comprehensive molecular profiling identifies actionable biomarkers for patients from Thailand and the United Arab Emirates with advanced malignancies

**DOI:** 10.3389/fonc.2024.1374087

**Published:** 2024-05-10

**Authors:** Shaheenah Dawood, Vasanti Natarajan, Pongwut Danchaivijitr

**Affiliations:** ^1^ Mohammed Bin Rashid University of Medicine and Health Sciences, Dubai, United Arab Emirates; ^2^ Oncology Department, Mediclinic City Hospital, Dubai, United Arab Emirates; ^3^ Global Medical Affairs, Caris Life Sciences, Basel, Switzerland; ^4^ Division of Medical Oncology, Department of Medicine, Faculty of Medicine, Siriraj Hospital, Mahidol University, Bangkok, Thailand

**Keywords:** next-generation sequencing, RNA fusions, cancer molecular profiling, cancer biomarkers, tumor mutational burden, immune checkpoint inhibitors, HER2 alteration

## Abstract

**Background:**

Comprehensive molecular profiling of tissue samples that can help guide therapy management is not widely available across the globe.

**Methods:**

Comprehensive molecular profiling through Caris Molecular Intelligence involves the analysis of DNA through next-generation sequencing, chromogenic or fluorescent in situ hybridization, pyrosequencing, and copy number alterations; RNA through whole-transcriptome sequencing and multiplex PCR of RNA; and protein through immunohistochemistry.

**Results:**

Here we describe the experience of molecular profiling of tumor tissue samples from patients diagnosed with advanced solid tumors and treated in two countries, the United Arab Emirates and Thailand. Tumor cancer cases submitted to Caris Life Sciences (Phoenix, Arizona, USA) for molecular profiling from the UAE and Thailand were retrospectively analyzed (data accessed between 2019 and 2020) for their molecular alterations and clinical biomarkers, without regard to ethnicity. A total of 451 samples from 35 distinct types of advanced cancers were examined for mutations, amplifications, overexpression, exon copy number alterations, microsatellite instability, deficient mismatch repair, tumor mutational burden, and fusions. Interrogating each step of the biological pathway, from DNA to RNA to distinct protein, identified an alteration with an associated therapy for 75% of these tumor samples. The most common alterations identified included elevated PDL-1 that can be targeted with an immune checkpoint inhibitors and amplification of HER2 for which a variety of anti HER2 therapies are available.

**Conclusion:**

Comprehensive molecular profiling in patients with advanced malignancies can help optimize therapeutic management allowing for improved prognostic outcome.

## 1 Introduction

Comprehensive molecular profiling has been empowered by rapid advances in molecular technologies examining DNA, RNA, and proteins. Through comprehensive molecular profiling, disease biomarkers can be identified and used to expand patients’ treatment options by revealing effective targeted therapies in what became known as precision medicine. When explored, molecular markers have been shown to carry clinical benefit in several therapeutic areas, such as infectious diseases, cardiovascular diseases, neurodegenerative disorders and most notably, malignancies (1–4).

In oncology, trends in molecular profiling have been increasingly positive as the list of approved biomarker matching targeted drugs continues to grow and new biomarkers continue to be identified. Several molecular markers have been detected in various cancer types that can be targeted with a molecular-matched therapy such as NTRK inhibitors, KRAS G12C inhibitors, and immune checkpoint inhibitors, to name a few. Other gene or protein alterations guiding treatment decisions are ALK, BCL-2, BRAF, BRCA, HER2, EGFR, and RAS mutations, among others (5). Molecular-guided treatment approaches thus allow treatment personalization and targeted cancer management, which was reflected in remarkable improvements in non-small cell lung cancer (NSCLC) outcomes (6, 7). It has become evident that the use of comprehensive molecular profiling to identify various actionable markers carries clinical benefit for cancer patients and improves overall survival (8, 9). Tissue-agnostic clinical trials have thus risen to prominence, constituting a noteworthy paradigm shift in both precision medicine and cancer treatment away from site-driven management (10). As of April 2024, seven therapies have been approved by the US FDA for tissue agnostic indications and include pembrolizumab [microsatellite instability and tumor mutational burden (TMB) ≥10 mutations/megabase (mut/Mb)] and larotrectinib/entrectinib (NTRK fusions) (11). This list is expected to grow as other agnostic biomarkers such as RET are currently being explored in clinical trials. Previous work has examined the relationships between biomarkers and cancer therapeutics, such as immune checkpoint inhibitors (12). However, comprehensive analyses remain necessary to elucidate the interplay between various biomarkers and different cancer types across molecular function levels, including DNA, RNA and proteins.

The use of molecular profiling is rapidly increasing in developed countries, but its systematic adoption remains quite limited around the globe. Molecular profiling relies heavily on next-generation sequencing (NGS), which is not readily available in several less industrialized regions. This study included samples from patients with 35 different types of advanced cancer from the United Arab Emirates (UAE) in the Middle East and Thailand in Asia. Here, we tested DNA, RNA, and protein to comprehensively seek targetable biomarkers. We aim to summarize the experience of these two underreported countries using a CLIA-certified diagnostic service.

## 2 Materials and methods

### 2.1 Tumor samples

Tumor cancer cases submitted to Caris Life Sciences (Phoenix, Arizona, USA) for molecular profiling were retrospectively analyzed (data accessed between 2019 and 2020) for their molecular alterations and clinical biomarkers. In total, 451 individual patient tumor samples were received and analyzed by Caris Life Science. The samples were simply sourced from two separate geographical regions (the UAE and Thailand); sub-analysis by ethnicity was not targeted. Authors had no access to information that could identify individual participants during or after data collection. Caris Molecular Intelligence includes NGS, whole-transcriptome sequencing (WTS), and relevant immunohistochemistry (IHC).

Formalin-fixed paraffin-embedded (FFPE) samples were sent for analysis from treating physicians from Thailand and UAE. The tissue diagnoses were submitted based on pathologic assessment of physicians who requested the assays and were further verified by a board-certified oncological pathologist at the Caris laboratory. Disease classification was then determined based on assessment of the submitted tissue along with associated clinical documentation. Age, gender, and specimen source information were available for analysis but demographic and patient clinical information were unavailable. Formalin-fixed paraffin-embedded (FFPE) tissue specimens were analyzed using NGS, IHC, chromogenic or fluorescent in situ hybridization (CISH/FISH), and/or pyrosequencing.

### 2.2 DNA next-generation sequencing

NGS was performed on genomic DNA isolated from FFPE tumor samples using the NextSeq platform (Illumina, Inc., San Diego, CA). Prior to molecular testing, tumor enrichment was achieved by harvesting targeted tissue using manual microdissection techniques. Matched normal tissue was not sequenced. A custom-designed SureSelect XT assay was used to enrich 592 whole-gene targets (Agilent Technologies, Santa Clara, CA). All variants were detected with > 99% confidence based on allele frequency and amplicon coverage, with an average sequencing depth of coverage of > 500-fold and an analytic sensitivity of 5%. Genetic variants identified were interpreted by board-certified molecular geneticists and categorized as ‘pathogenic,’ ‘presumed pathogenic,’ ‘variant of unknown significance,’ ‘presumed benign,’ or ‘benign,’ according to the American College of Medical Genetics and Genomics (ACMG) standards. When assessing mutation frequencies of individual genes, ‘pathogenic,’ and ‘presumed pathogenic’ were counted as mutations while ‘benign’, ‘presumed benign’ variants and ‘variants of unknown significance’ were excluded.

### 2.3 Copy number alteration

The copy number alteration (CNA) of each exon was determined in DNA by calculating the average depth of the sample along with the sequencing depth of each exon and comparing this calculated result to a pre-calibrated value.

### 2.4 Microsatellite instability and mismatch repair

Microsatellite instability (MSI) was examined using over 7,000 target microsatellite loci and comparing those with the reference genome hg19 from the University of California, Santa Cruz (UCSC) Genome Browser database (13). The status was defined as MSI-high (MSI-H) or MSI-low/microsatellite-stable (MSS). The number of microsatellite loci that were altered by somatic insertion or deletion were counted for each sample. Only insertions or deletions that increased or decreased the number of repeats were considered. Genomic variants in the microsatellite loci were detected using the same depth and frequency criteria as used for mutation detection. MSI-NGS results were compared with results from over 2,000 matching clinical cases analyzed with traditional PCR-based methods (14). In order to generate a sensitivity of >95% and specificity of >90%, the threshold to determine MSI by NGS was determined to be 46 or more loci with insertions or deletions. MSI and mismatch repair (MMR) status were determined with a combination of test platforms that included fragment analysis (FA; Promega, Madison, WI), IHC (MLH1: M1 antibody; MSH2: G2191129 antibody; MSH6: 44 antibody; and PMS2: EPR3947 antibody [Ventana Medical Systems, Inc., Tucson, AZ, USA]), and NGS. NGS used the NextSeq platform, which has 7,000 target microsatellite loci that were examined and compared to the reference genome hg19 from the University of California. The status of the tumor regarding MSI and MMR was determined first with IHC, then with FA, and finally with NGS if necessary.

### 2.5 Tumor mutational burden

TMB was measured by counting all non-synonymous missense, nonsense, in-frame insertions, in-frame deletions, and frameshift mutations found per tumor that had not been previously described as germline alterations in dbSNP151, Genome Aggregation Database (gnomAD) databases (15), or benign variants identified by Caris geneticists. A cutoff point of ≥ 10 mut/Mb was used based on the KEYNOTE-158 pembrolizumab trial (16), which showed that patients with a TMB of ≥ 10 mut/Mb across several tumor types had higher response rates than patients with a TMB of < 10 mut/Mb. Caris Life Sciences is a participant in the Friends of Cancer Research TMB Harmonization Project (17).

### 2.6 Fusion detection by gene panel and by whole-transcriptome sequencing

Gene fusions were detected by ArcherDx fusion assay (Archer FusionPlex Solid Tumor panel) on Illumina MiSeq platform or WTS (Agilent SureSelect Human All Exon V7 bait panel) on Illumina NovaSeq platform (Illumina, Inc., San Diego, CA). The formalin-fixed paraffin-embedded tumor samples underwent pathology review and were micro-dissected to enrich the tumor nuclei prior to mRNA extraction. For Archer assay, unidirectional gene-specific primers were used to enrich for target regions, followed by NGS (Illumina MiSeq platform). Targets included 52 genes, and the full list can be found at http://archerdx.com/fusionplex-assays/solid-tumor. For WTS, biotinylated RNA baits were hybridized to the synthesized and purified cDNA targets and the bait-target complexes were amplified in a post capture PCR reaction. The resultant libraries were quantified, normalized and the pooled libraries are denatured, diluted, and sequenced; the reference genome used was GRCh37/hg19.

### 2.7 In situ hybridization

Chromogenic in situ hybridization (CISH) was used for Her2/neu (INFORM HER2 Dual ISH DNA Probe Cocktail). Her2 test results were considered amplified if HER2/CEP17 ratio ≥ 2.0 with an average HER2 copy number ≥ 4.0 signals per cell; or if HER2/CEP17 ratio ≥ 2.0 with an average HER2 copy number < 4.0 signals/cell; or HER2/CEP17 ratio < 2.0 with an average HER2 copy number ≥ 6.0 signals/cell.

### 2.8 Pyrosequencing

MGMT promoter methylation was evaluated by pyrosequencing. DNA was extracted from FFPE tumor samples. The five CpG sites (CpGs 74-78) were analyzed by pyrosequencing. All DNA samples underwent a bisulfite treatment and were PCR amplified with primers specific for exon 1 of MGMT (GRCh37/hgl9 – chr10: 131,265,448- 131,265,560). The methylation status of PCR-amplified products was determined using the PyroMark system. Samples with < 7% methylation were considered negative, those with > 9% methylation were considered positive, and those with ≥ 7% and < 9% methylation were considered to equivocal results.

### 2.9 Associations between drugs and biomarkers

Associations between drugs and biomarkers were determined based on a combination of FDA guidelines, professional society guidelines and review and summary of clinical literatures.

### 2.10 Ethics considerations

This study was conducted using retrospective, de-identified clinical data submitted to Caris Life Sciences (Phoenix, Arizona, USA), and patient consent was not required for the processed specimens in Thailand. Ethical approval was obtained from the research ethics committee as per local regulations in the UAE.

## 3 Results

### 3.1 Specimen processing

**Figure 1:**
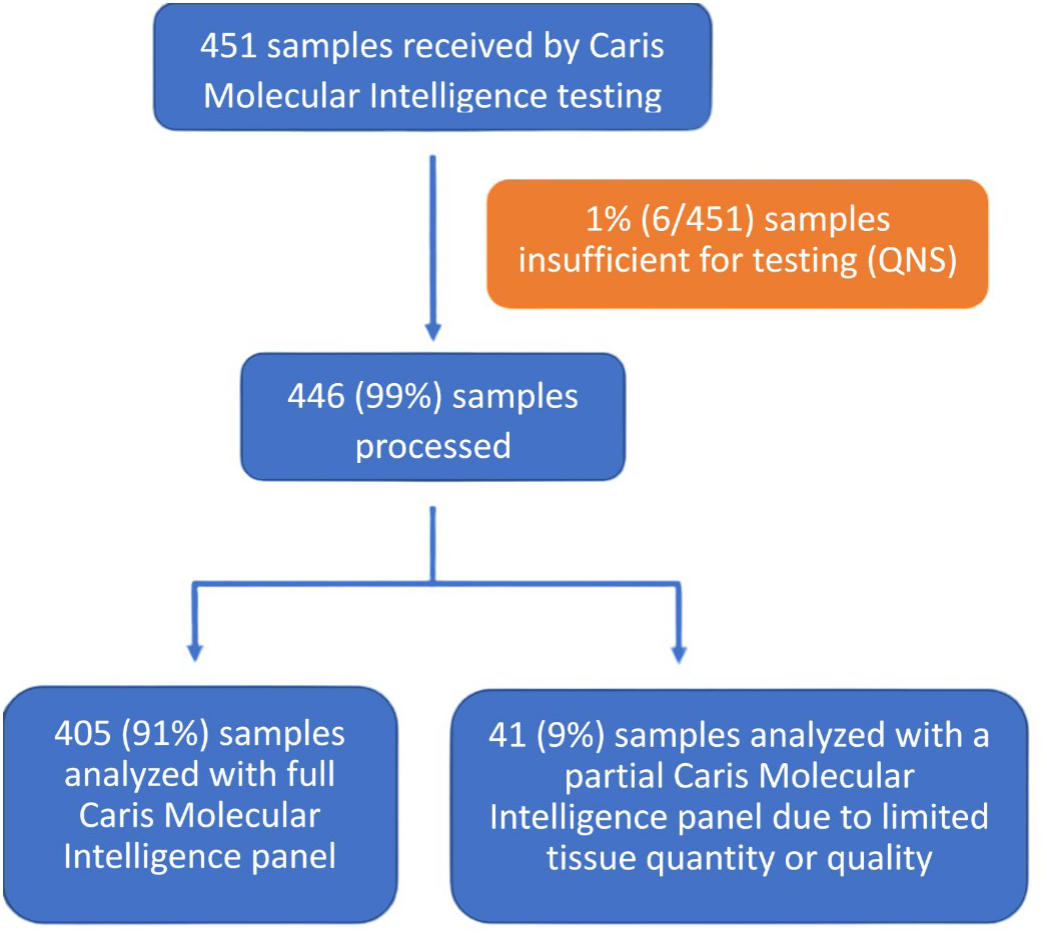
Specimen processing and overall clinical utility. CMI, Caris Molecular Intelligence; QNS, quantity not sufficient.

Of the 451 submitted for molecular testing, 446 (99%) were processed and 405 (91%) were sequenced successfully with all tests of the Caris Molecular Intelligence assays that were deemed relevant for the specific cancer type (**Figure 1**). Partial, rather than comprehensive molecular testing, was be performed on 41 samples (9%) due to tissue with limited quantities or poor quality. A total of 6 samples (1%) were not tested due to the samples having an insufficient quantity for analysis.

### 3.2 Cohort characteristics

**Figure 2:**
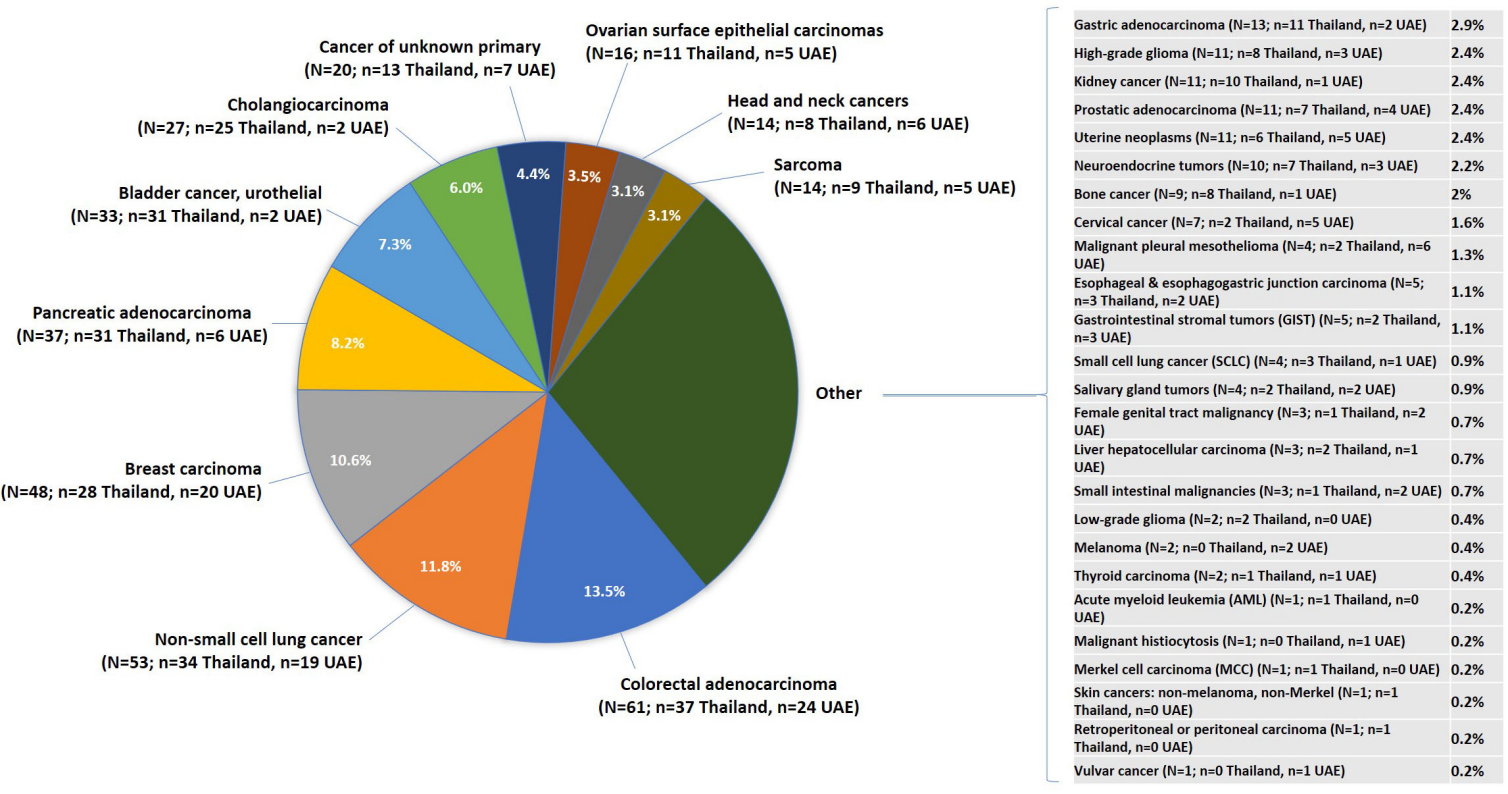
Breakdown of cancer types included in the study cohort. UAE, United Arab Emirates.

Caris Life Science received 451 individual patient tumor samples between 2019 and 2020 (**Figure 2**), which included 303 from Thailand and 148 from UAE. The median age of the patients was 59 years, with a range from 19 to over age 89 years, and the patients were 51% male and 49% female. A total of 35 distinct tumor types were included, with the most common cancers being colorectal adenocarcinoma (CRC; 13.5%), NSCLC (11.8%), and breast carcinoma (10.6%) in the overall cohort. Following CRC and NSCLC, pancreatic adenocarcinoma and bladder cancer, urothelial were the third most common in Thailand. Breast carcinoma was the most common cancer type in the UAE, followed by CRC and NSCLC.

### 3.3 Distribution and types of genomic alterations

**Figure 3:**
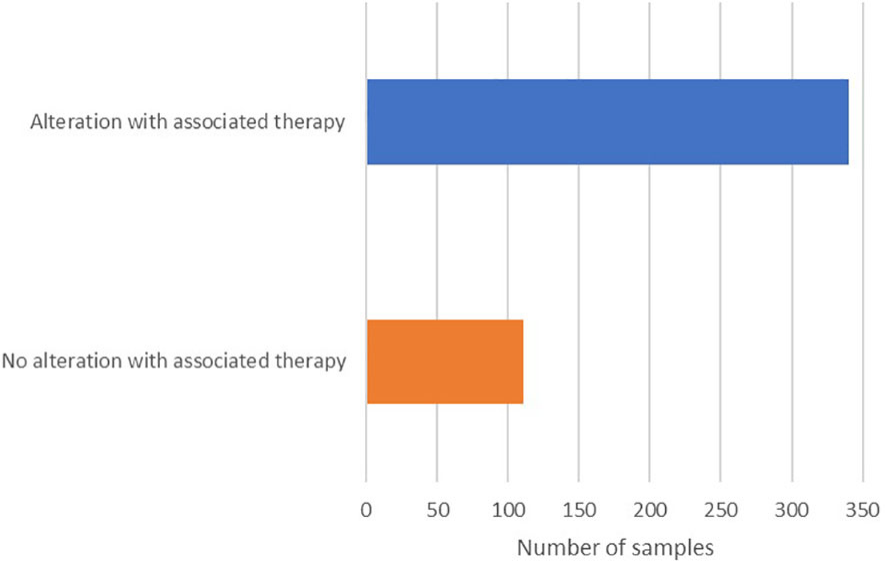
Patient samples with alterations with or without associated therapies.

Of the 451 samples profiled, 436 (97%) had at least one alteration detected (**Table 1**), and 340 (75%) had at least one alteration with an associated therapy (**Figure 3**). The frequency of pathogenic and likely-pathogenic genomic alterations varied widely across tumor types. DNA mutations and copy number amplifications were the most commonly observed events. Immunotherapy biomarkers, including elevated PD-L1 and TMB-high, were most common in NSCLC (PD-L1 ≥1% in 63%, ≥10% in 43%, and TMB-high in 17% among 53 samples), head and neck cancer (PD-L1 ≥1% in 100%, ≥10% in 77% among 14 samples), and in urothelial bladder cancer (PD-L1 ≥1% in 88% and TMB-high in 33% among 33 samples).

**Table 1:** Frequency of pathogenic alterations.

Cancer Type	N	Any alterations detected		≥1 alteration with an associated therapy		Checkpoint Inhibitor Biomarkers				Gene fusion	>1 DNA mutation detected	>1 DNA amplification detected
						TMB-H	≥ 1% PD-L1	≥ 10% PD-L1	dMMR/ MSI-H			
		N	%	n	%	%	%	%	%	%	%	%
Colorectal adenocarcinoma	61	59	97	39	64	7	5	2	3	0	93	39
NSCLC	53	52	98	48	91	17	63	43	0	16	87	32
Breast carcinoma	48	47	98	42	88	15	45	9	2	9	88	44
Pancreatic adenocarcinoma	37	33	89	26	70	0	29	10	3	3	70	24
Bladder cancer, urothelial	33	32	97	30	91	33	88	31	3	0	91	61
Cholangiocarcinoma	27	27	100	18	67	8	44	15	0	0	93	19
Cancer of unknown primary	20	20	100	17	85	17	63	37	0	16	85	30
Ovarian surface epithelial carcinomas	16	16	100	15	94	7	50	13	0	6	75	13
Head and neck cancers	14	14	100	13	93	0	100	77	0	0	79	21
Sarcoma	14	13	93	7	50	0	43	29	0	0	64	29
Gastric adenocarcinoma	13	13	100	9	69	10	54	15	0	0	62	62
Prostatic adenocarcinoma	11	9	82	7	64	29	22	11	11	44	45	18
High-grade glioma	11	10	91	8	73	11	50	30	10	20	55	36
Uterine neoplasms	11	10	91	7	64	10	18	9	10	0	55	27
Kidney cancer	11	10	91	5	45	13	56	33	0	0	64	9
Neuroendocrine tumors	10	10	100	7	70	0	50	10	0	0	100	40
Bone cancer	9	9	100	4	44	0	44	11	0	33	33	22
Cervical cancer	7	7	100	6	86	20	71	43	0	0	86	43
Malignant pleural mesothelioma	6	6	100	3	50	0	50	0	0	0	100	0
Esophageal and esophagogastric junction	5	5	100	5	100	0	100	40	0	0	100	60
Gastrointestinal stromal tumors (GIST)	5	5	100	2	40	0	50	25	0	0	60	0
SCLC	4	4	100	2	50	33	50	0	0	0	50	0
Salivary gland tumors	4	4	100	2	50	0	0	0	0	50	75	0
Female genital tract malignancy	3	3	100	3	100	33	33	0	33	0	100	33
Liver hepatocellular carcinoma	3	3	100	0	0	0	0	0	0	0	67	0
Small intestinal malignancies	3	3	100	3	100	0	0	0	0	0	100	33
Melanoma	2	2	100	2	100	50	0	0	0	50	100	50
Low-grade glioma	2	2	100	2	100	0	0	0	0	0	100	0
Thyroid carcinoma	2	2	100	2	100	0	50	0	0	0	100	0
Acute myeloid leukemia (AML)	1	1	100	1	100	0	0	0	0	0	100	100
Malignant histiocytosis	1	1	100	1	100	0	100	0	0	100	0	0
Merkel cell carcinoma (MCC)	1	1	100	1	100	0	0	0	0	0	100	0
Skin cancers: non-melanoma, non-Merkel cell	1	1	100	1	100	100	100	100	100	0	100	0
Retroperitoneal or peritoneal carcinoma	1	1	100	1	100	0	0	0	0	0	100	0
Vulvar cancer	1	1	100	1	100	0	100	100	0	0	100	0

dMMR, Mismatch repair deficient; MSI-H, Microsatellite instability-high; NSCLC, Non-small cell lung cancer; PD-L1, Programmed death-ligand 1; SCLC, Small cell lung cancer; TMB-H, Tumor mutational burden-high.

Biomarkers with potential therapy associations were identified on a transcriptomic and protein level, as appropriate (**Table 2**); Biomarker alterations associated with a therapy were identified 596 times by NGS, 389 by IHC, and 12 by WTS. Additionally, specific alterations were identified 12 times by CISH for HER2, 5 by pyrosequencing for MGMT-methylation, 2 by CNA for MET alterations, and 10 by the combination of IHC, FA, and, if necessary, NGS for dMMR/MSI-H.

**Table 2:** Biomarker alterations associated with therapies.

Platform	Protein or gene	Biomarker	Associated therapies	Total, N^*^	Negative, n	Positive, n	Incidence, %
IHC	ALK	ALK	ALKi	39	36	3	7.7
WTS	ALK			406	403	3	0.7
IHC	AR	AR	Anti-androgen	60	31	29	48.3
NGS	BRAF	BRAF	BRAFi/MEKi	401	394	7	1.7
NGS	BRCA1	BRCA1/2	PARPi, platinum	396	387	9	2.3
NGS	BRCA2			395	383	12	3.0
IHC	EGFR	EGFR	EGFR TKI	403	384	19	4.7
IHC	ER	ER	Hormone therapy	71	32	39	54.9
IHC	HER2/Neu	ERBB2 (HER2)	HER2-targeted agents	141	131	10	7.1
CISH	HER2			55	43	12	21.8
NGS	ERBB2			401	393	8	2.0
CNA	ERBB2			400	385	15	3.8
NGS	ESR1	ESR1	Combination endocrine therapy & targeted therapy	403	398	5	1.2
NGS	FGFR2	FGFR2	FGFRi	401	398	3	0.7
WTS	FGFR2			406	405	1	0.2
NGS	FGFR3	FGFR3		392	387	5	1.3
WTS	FGFR3			406	404	2	0.5
NGS	IDH1	IDH1	IDHi	404	397	7	1.7
NGS	KIT	KIT	TKI	403	401	2	0.5
NGS	KRAS	KRAS	KRASi; resistance to therapies	404	315	89	22.0
NGS	MET	MET	METi	402	400	2	0.5
CNA	MET			401	399	2	0.5
WTS	Exon14skip			405	403	2	0.5
Pyro- sequencing	MGMT-Me	MGMT Me	Temozolomide	9	4	5	55.6
IHC FA NGS	dMMR/MSI-H	dMMR/ MSI-H	Checkpoint inhibitors	433	423	10	2.3
WTS	NTRK3	NTRK3	NTRKi	405	404	1	0.2
NGS	PALB2	PALB2	PARPi	402	400	2	0.5
IHC	PD-L1 ≥1%	PD-L1	Checkpoint inhibitors	394	214	180	45.7
IHC	PD-L1 ≥10%			394	310	84	21.3
NGS	PIK3CA	PIK3CA	PI3Ki	403	349	54	13.4
NGS	POLE	POLE	Checkpoint inhibitors	399	398	1	0.3
IHC	PR	PR	Hormone therapy	65	40	25	38.5
NGS	RET	RET	RETi	401	400	1	0.2
WTS	RET			405	403	2	0.5
NGS	TMB-H	TMB	Checkpoint inhibitors	45	338	383	11.7

^*^N values do not include results where testing results were indeterminate due low coverage.

CISH, Chromogrenic in situ hybridization; CNA, copy number alternation; dMMR, mismatch repair deficient; FA, Fragment analysis; i, inhibitor (for example, ALKi=ALK inhibitor); IHC, immunohistochemisty; MSI-H, microsatellite instability-high; NGS, Next generation sequencing; TMB-H, tumor mutational burden-high; WTS, Whole transcriptome sequencing.

Observed protein alterations included positive expression of ER by IHC (55%, n = 39 of 71 samples), PR by IHC (39%, n = 25 of 65 samples), and AR (48%, n = 29 of 60 samples). NGS identified mutations as commonly occurring in TP53 (60% of 385 samples tested), KRAS (22% of 404 samples tested), APC (13% of 398 samples tested), PIK3CA (13% of 403 samples tested), and KMT2D (9% of 366 samples tested) across the study cohort. CISH identified HER2 amplification in 12 of the 55 samples tested, plus IHC identified HER2 amplification in 10 of 141 samples tested, while CNA identified alterations of HER2 copy numbers in 15 of 400 samples tested, and NGS identified DNA alterations of HER2 in 10 of 141 samples tested, which included 8 samples with HER2 mutations. Actionable BRCA1/2 mutations detected by NGS were not limited to breast and ovarian cancer, but were observed in several tumor types (e.g. colorectal cancer, glioblastoma, head and neck cancer, pancreatic adenocarcinoma, prostate cancer). IHC was also performed in a subset of patients in a reflex situation to detect EGFR in the absence of DNA.

A total of 24 aberrations in RNA were identified by RNA sequencing or by WTS (**Table 3**). Among the six fusions identified in breast carcinoma samples of the 38 samples examined, three involved NOTCH2, and one each involved FGFR3, NTRK3, and BRAF. Among 53 NSCLC samples examined for fusions, three had fusions involving ALK, two involved RET, and one involved ROS1. Among the 6 fusions identified in NSCLC samples, three involved ALK and two involved RET. Among the nine bone cancer samples examined, three fusions involving EWSR1 were identified. Among 20 cancers of unknown primary examined, two had the CLDN18:ARHGAP26 fusion, which has been reported in gastric carcinomas and may suggest the origin of the unknown primary tumor (18, 19).

**Table 3:** Gene fusions identified across tumor types.

Tumor type	Fusion frequency			N	Associated tx
	%	n/N	Fusion		
Breast carcinoma	16	6/38	FGFR3:TACC3	1	FGFRi
			CALM2:NOTCH2	1	
			PCMTD1:NOTCH2	1	
			TXNL4B:NOTCH2	1	
			PML : NTRK3	1	NTRKi
			GTF2IRD2B:BRAF	1	
NSCLC	11	6/53	EML4:ALK	3	ALKi
			CCDC6:RET	1	RETi
			CCDC186:RET	1	RETi
			CD74:ROS1	1	
Bone cancer	33	3/9	EWSR1:ETV1	1	
			EWSR1:FLI1	1	
			EWSR1:NR4A3	1	
Cancer of unknown primary	15	3/20	CLDN18:ARHGAP26	2	
			CD74:ROS1	1	
High-grade glioma	18	2/11	FGFR3:TACC3	1	FGFRi
			EGFRvIII	1	
Melanoma	50	1/2	KIAA1549:BRAF	1	
Ovarian	6	1/16	NSD3:FGFR1	1	
Pancreatic	3	1/37	FGFR2:INA	1	FGFRi
Prostate adenocarcinoma	36	4/11	TMPRSS2:ERG	3	
			NDRG1:ETV4	1	
Salivary gland tumor	50	2/4	MYB : NFIB	2	
Sarcoma (malignant histiocytosis)	7	1/14	ZKSCAN1:BRAF	1	

i, inhibitor (for example, ALKi=ALK inhibitor); NSCLC, non-small cell lung cancer; tx, treatment.

## 4 Discussion

Challenging clinical cases, such as advanced tumors, relapsed/refractory tumors and cancers of unknown origin, push the boundaries of traditional cancer treatment. Tumor-agnostic therapy bypasses the inherent limitation of treating malignancies based on pathologic classification and tissue of origin through the means of a targeted approach. Molecular screening has been shown to be both clinically feasible and effective in revealing actionable alterations and/or resistance-conferring mutations (20). Tissue-agnostic therapy allows early treatment personalization based on genetic mutations and biomarkers and improves clinical outcomes (e.g. treatment response, overall and progression-free survival) (8, 9). This therapeutic advantage was illustrated in the present study; biomarkers with therapy associations were identified among 75% of samples subjected to comprehensive exome and transcriptome analysis. Notably, 85% of samples from cancer of unknown primary had at least one alteration associated with a therapy identified. In the absence of a clear standard treatment paradigm for cancer of unknown primary, comprehensive molecular profiling clearly expands treatment options beyond systemic therapy or palliative care.

Elevated levels of PD-L1 and amplification of HER2 were frequently observed among the targetable alterations identified in this study. Once high levels of PD-L1 expression are detected, targeted therapy through immune checkpoint inhibitors can be used. In a similar vein, HER2-targeting drugs can be commenced in case of identified HER2 amplification. Targetable HER2 alterations include *ERBB2* mutations, amplifications, and changes in HER2 protein expression. The oncogenic potential of HER2 alterations is variable and dependent on primary tumor histology (21). Targeting HER2 has expanded from treating the overexpression of HER2 in breast cancer along to other malignancies. Based on this, HER2 inhibitors could be considered for patients in the study sample with actionable HER2 amplification in their tumors, which included colorectal cancer, stomach cancer and esophageal cancer. Similarly, the PD-L1-directed therapy pembrolizumab provides clinical benefit in patients with advanced cancers of different origins that are heavily pre-treated, have metastatic breast cancer, and high TMB (22). PD-L1 expression ensures candidacy for PD-L1 inhibitors across a wide range of tumor types, such as advanced/metastatic NSCLC, head and neck squamous cell carcinoma, advanced/metastatic urothelial carcinoma, and gastric or gastroesophageal junction adenocarcinoma (23). Both PD-L1 expression and TMB were found to be a promising biomarker of survival and response to precision immunotherapy in NSCLC (24). The predictive efficacy of TMB has recently risen to prominence, with demonstrated prognostic role in malignancies such as NSCLC and CRC (25–28). TMB reflects the total number of somatic mutations per coding area of a tumor genome and shows variability according to tumor type as well as individual patients (29–31). Despite that, TMB seems promising in predicting pathological response to targeted therapy such as immune checkpoint inhibitors, as well as resistance to treatment (25–28). That being said, more prospective trials are needed to fully confirm these associations with treatment efficacy and clinical outcomes.

It is therefore clear, both on a theoretical and clinical level, that an evidence-based approach to identify biomarker-drug associations has the potential to transform patient outcomes. Even though individual markers have low prevalence, comprehensive molecular testing makes use of genomic, transcriptomic, and proteomic insights from the tumor tissue to maximize the chance for target identification. Some examples from the present study show the opportunities provided by molecular profiling in guiding and personalizing cancer therapy even for less common genetic alterations. One of the breast carcinoma samples tested exhibited a fusion in the neurotrophic receptor tyrosine kinase 3 (*NTRK3)* gene. Fusions of the *NTRK* gene are clinically actionable, with histology-agnostic responses to larotrectinib and entrectinib occurring in both adult and pediatric patients with cancers exhibiting NTRK fusions (32). Patients whose locally advanced or metastatic urothelial carcinoma carries *FGFR3-TACC3* fusions or certain genetic alterations in *FGFR3* or *FGFR2* can be treated with erdafitinib, with an overall response rate of almost 50% (33). Additional agents targeting multiple FGFRs or selectively targeting FGFR4 are in development (34). Efforts are underway to expand the use of FGFR-targeting molecules to earlier disease stages and other solid tumors. As more evidence emerges, this targeted therapy could prove valuable for patients carrying genetic mutations or fusions in FGFR, such as those observed in our study with high-grade glioma, ovarian cancer and pancreatic cancer.

This study is limited by its lack of follow-up data on the number of patients who received the suggested therapies and on their respective clinical outcomes. Regardless, it is expected that clinical benefit follows the use of therapies identified by Caris Molecular Intelligence. This agrees with recent publications that indicate clinical benefit occurred in 37% of patients who received the suggested therapy (8) and that overall survival was significantly improved for patients who received matched therapies compared with those who received unmatched therapies (9).

It is therefore essential to improve access to molecular profiling across the globe. Broad molecular profiling continues to face significant challenges, such as lack of infrastructure, lack of funding and low awareness; this in turn contributes to the poor accessibility of genomic testing and the limited understanding of its benefit and cost effectiveness among clinical oncologists and stakeholders (35, 36). Sporadic drug availability also hinders the implementation of molecular profile-based precision medicine in clinical practice (37). Policy changes are imperative for comprehensive multidisciplinary cancer management and should aim to promote equitable access to comprehensive molecular profiling, while also ensuring that both targeted therapies and adequate clinical expertise are available (38, 39).

## 5 Conclusions

Comprehensive molecular profiling identified biomarkers associated with therapies for 75% of the samples tested in this population of patients from countries where molecular profiling is not widely used. Molecular profiling offers the majority of these patients an option for a therapy that is targeted to their tumor. This is expected to lead to improved clinical outcomes. Large prospective trials remain necessary to confirm these findings and demonstrate the benefit of integrating comprehensive molecular profiling into clinical practice, particularly in developing countries with limited molecular resources.

## Data availability statement

The datasets presented in this article are not readily available because of the terms of use of Caris Molecular Intelligence. Requests to access the datasets should be directed to the corresponding author.

## Ethics statement

The studies involving humans were approved by Dubai Scientific Research Ethics Committee (DSREC), Dubai Health Authority Dubai, UAE. The studies were conducted in accordance with the local legislation and institutional requirements. The ethics committee/institutional review board waived the requirement of written informed consent for participation from the participants or the participants’ legal guardians/next of kin because the study used retrospective, de-identified clinical data submitted to Caris Life Sciences (Phoenix, Arizona, USA). Ethical approval was waived in Thailand.

## Author contributions

SD: Conceptualization, Investigation, Supervision, Writing – review & editing. VN: Conceptualization, Funding acquisition, Project administration, Writing – review & editing. PD: Conceptualization, Formal analysis, Investigation, Methodology, Resources, Supervision, Writing – original draft, Writing – review & editing.
